# Editorial: COVID-19 booster vaccination: increasing immunity against life-threatening infection

**DOI:** 10.3389/fpubh.2023.1342118

**Published:** 2024-01-09

**Authors:** Ritthideach Yorsaeng, Kamolthip Atsawawaranunt, Abanoub Riad

**Affiliations:** ^1^Center of Excellence in Clinical Virology, Department of Pediatrics, Faculty of Medicine, Chulalongkorn University, Bangkok, Thailand; ^2^King Chulalongkorn Memorial Hospital, Thai Red Cross Society, Bangkok, Thailand; ^3^Institute for Urban Disease Control and Prevention, Department of Disease Control, Ministry of Public Health, Bangkok, Thailand; ^4^Department of Public Health, Faculty of Medicine, Masaryk University, Brno, Czechia; ^5^Institute of Health Information and Statistics of the Czech Republic (IHIS-CR), Prague, Czechia; ^6^Czech National Centre for Evidence-Based Healthcare and Knowledge Translation (Cochrane Czech Republic, Czech EBHC: JBI Center of Excellence, Masaryk University GRADE Centre), Faculty of Medicine, Institute of Biostatistics and Analyses, Masaryk University, Brno, Czechia

**Keywords:** SARS-CoV-2, COVID-19, vaccines, booster vaccination, immunity, immune response, COVID-19 vaccination

The emergence of Severe Acute Respiratory Syndrome Coronavirus 2 (SARS-CoV-2) has caused the global transmission of Coronavirus disease 2019 (COVID-19) and continues to evolve. COVID-19 vaccines were rapidly developed within a year of the disease's emergence. In the early stages of the pandemic, COVID-19 vaccines were designed based on the related ancestral (wild-type) strain and were typically administered in two shots for full priming vaccination. They proved effective against severe infections but did not provide complete protection against symptomatic infections (1). Breakthrough infections commonly occur even after a full priming vaccination (1, 2). The main reasons for this are waning immunity (3) and the emergence of newly evolved variants of concern (VOCs), such as Delta (B.1.617.2) and Omicron (B.1.1.529), which have higher contagiousness and altered amino acid sequences that evade immunity (2–4). However, vaccines still offer protection against life-threatening infections and reduce the likelihood of long-term sequelae (long COVID-19) (1). Furthermore, high-risk groups, such as older adults (5), those with underlying medical conditions (6), obese individuals (7), immunocompromised individuals, solid organ transplant recipients (8), and oncology patients, including the recipients of immunotherapy or chemotherapy (9, 10), are at greater risk of life-threatening infection or mortality due to insufficient immune response compared to healthy individuals. Given the waning immunity and circulation of emerging VOCs, and the vulnerability of high-risk groups, it is evident that full priming vaccination may not provide sufficient protection against the widespread global spread of the disease.

During the crisis and with limited resources, booster vaccinations emerged as a potential strategy to tackle VOCs and served as a “makeshift” approach when reliable drugs and vaccines were not readily available. At the time, neither the second generation (e.g., bivalent) nor beyond (e.g., XBB monovalent) had been introduced. Boosters had the potential to significantly enhance immunity through an anamnestic response, addressing the issue of waning immunity, restoring reduced effectiveness, and prolonging high levels of immunity. This approach aimed to reduce the viral load in breakthrough infections (2, 11), consequently reducing the likelihood of disease transmission. Immunity levels were closely associated with vaccine efficacy/effectiveness, particularly in protecting against life-threatening infections (12), making the maintenance of high immunity crucial during the crisis. Studies indicated that booster vaccinations reduced the rate of COVID-19 cases, severe illness, and mortality compared to those who received only the initial vaccination (13). Moreover, it was observed that the use of the inactivated platform with an “old-fashioned” adjuvant (aluminum-based) resulted in lower antibody levels compared to other platforms (14). In response, the adenoviral vector platform was considered a potential booster, demonstrating high efficacy against the Delta variant (15). Similarly, mRNA or protein subunit platforms have shown potential for enhancing immunity (16, 17), even in fractional dose vaccination (18). While the increased immunity from the ancestral strain vaccine remained effective against the Omicron variant, it was notably less effective than against the ancestral strain (19). Conversely, booster vaccine effectiveness was anticipated to be higher and more durable compared to relying solely on full priming vaccination, maintaining efficacy against VOCs (20). In particular, heterologous adenoviral vectors following mRNA booster vaccines have shown promise in reducing severe disease, even in immunocompromised and high-risk individuals and older adults (13, 21). Additionally, using the same platform (mRNA) with different vaccines provided better protection against symptomatic and severe infections than using the same vaccine (22). Several countries have endorsed booster vaccinations as an effective strategy to reinforce and sustain immunity against COVID-19. However, limited data are available to comprehensively explore the outcomes of COVID-19 booster vaccinations, including adverse events following immunization (AEFI). Therefore, this Research Topic aims to focus on the effects of COVID-19 booster vaccinations by examining evidence from animal models, clinical trials, real-world observations, and systematic reviews.

We have received a total of 41 manuscripts relevant to this Research Topic, of which 28 articles met the eligibility criteria for publication in three sections of Frontiers in Public Health/Infectious Diseases: Epidemiology and Prevention, Frontiers in Medicine/Infectious Diseases: Pathogenesis and Therapy, and Frontiers in Immunology/Vaccines and Molecular Therapeutics. These articles are of various types and include 21 original articles, 4 systematic reviews, 2 brief research reports, and 1 clinical trial article.

In preclinical studies using mouse models, researchers explored potential SARS-CoV-2 vaccine candidates. Qin et al. developed a universal mRNA vaccine platform containing Delta or Omicron variant spikes or a multi-T cell epitope (MTE). Vaccines with only the MTE protected mice from lethal Delta variant challenges. Combining spike-specific variants and the MTE showed promise as a universal SARS-CoV-2 vaccine. Zhou Y. et al. designed a live attenuated *Pseudomonas aeruginosa* bacterial vector expressing the SARS-CoV-2 RBD protein through a bacterial type III secretion system (injectisome) with candidate plasmids of wild-type, Delta, or Omicron BA.1. The complex bacterial nanomachine stimulated mucosal immunity via the intranasal route of administration, and vaccine safety was evaluated based on lung pathology. The results showed that the serum elicited good antibody and T-cell responses. Li et al. evaluated various adenoviral vector and/or mRNA platforms as prime-boost strategies in the intramuscular and/or intranasal route. The vectors were encoded with the wild-type or Beta variant spike gene; mRNAs were encoded with the wild-type or Omicron variant spike gene. This study assessed cellular immune responses, neutralizing different variants and subvariants. Interestingly, primary vaccination with an intranasal adenoviral vector encoding the Beta spike gene, followed by intramuscular mRNA encoding the Omicron spike gene, induced a broader spectrum and stronger IgA and neutralized against variants. This suggests that heterologous strategies between platforms, routes, and antigens could generate broad-spectrum immunity and enhance neutralizing capacity.

A brief research report from Wu et al. reported on the safety and immunogenicity of a full priming inactivated vaccine compared with a homologous prime-boost protein subunit vaccine in chronic hepatitis B patients. The results showed a safe and high seropositive rate. The seropositive rate was lower in patients with cirrhosis than in patients without cirrhosis. Another brief research report by Perico et al. assessed the humoral and cellular responses in healthcare workers, both naïve and convalescent subjects. This study was a long-term follow-up for 12 months, 9 months after the full priming, and followed by 3 months after the booster. Hybrid immunity resulted in significantly higher antibody levels than naïve individuals. The humoral response levels are linked to specific memory B cells. The study suggested that boosters may enhance the immune response, particularly in maintaining antibodies, especially in naïve subjects. The cohort studies focus on healthy participants, mainly on immunogenicity. Leung et al. conducted a clinical trial comparing humoral and cellular immunity, including tests specific for the Omicron variant of homologous inactivated prime-boost vaccination in healthy adolescents and healthy adults. The reactogenicity was mild. Immunogenicity outcomes in adolescents were higher than in adults, with neutralizing and cellular immunity potentially protective against the Omicron BA.1 subvariant. Hyun et al. compared the immunogenicity of Ad26.COV2.S or mRNA boosters in full priming of Ad26.COV2.S (single-shot). This study showed results for both humoral and cellular immunity. Neutralizing antibodies were enormously increased against wild-type but were lower in Omicron BA.5, and Omicron BA.1 elicited the lowest level compared with the others. IgG anti-RBD and specific interferon-γ were significantly increased after vaccination, except that the Ad26.COV2.S booster group did not increase the interferon-γ. This study suggested that the heterologous prime-boost adenoviral vector and mRNA platforms could increase immunity more than the homologous prime-boost adenoviral vector platform. Additional boosters may be helpful to increase immunity against the Omicron sub-variants. Lozano-Rodríguez et al. evaluated the overall immunological responses in naïve and convalescent participants vaccinated with heterologous prime-boost mRNAs (BNT162b2 and mRNA-1273) vaccination with long-term follow-up over 1 year. Humoral responses increased substantially but waned after 6 months, while T-cell responses remained stable. The immune response in a convalescent group was higher than in naïve participants. However, immunity against the Omicron BA.1 sub-variant was lower than the wild-type in both groups. Huang et al. investigated T-cell responses from homologous inactivated prime-boost vaccination. The booster enhances and broadens T-cell responses against SARS-CoV-2 spike and non-spike antigens from wild-type, Delta, and Omicron BA.1. Hosseinian et al. assessed the antibody profile of participants who received mRNA boosters at 6-month follow-up. Neutralizing antibodies have reduced activity against Delta and substantially reduced activity against Omicron variants, particularly the BA.1 sub-variant, compared to the BA.2 sub-variant. Booster antibody levels remained significantly higher than pre-booster, even with waning antibody levels at 6 months. Severe systemic side effects were linked to higher antibody levels and could persist for several months. Antigen microarray characterization revealed little cross-reactivity between SARS-CoV-2 and other coronaviruses or influenza viruses. The study suggested that breakthrough infections may be driven by specific antigens from new variants rather than waning immunity. Dou et al. performed a pilot-scale single-cell sequencing analysis of the inactivated vaccine recipients. The inactivated vaccine promoted T cell proliferation, T cell receptor clone amplification, and diversity. This finding showed that the booster significantly enhanced CD8^+^ mucosal-associated invariant T (MAIT) cell proliferation and differentiation, and KLRD1 gene expression in NK cells was significantly higher. This study suggests that an inactivated vaccine platform could stimulate an early adaptive T cell response against the virus.

Four cohort studies focus on the immunocompromised host. Collectively, these studies underscore the impact of booster strategies on enhancing immunity in immunocompromised individuals and highlight the complexities and challenges of achieving adequate responses in this population. Wang et al. conducted a clinical trial comparing the effects of shorter (3 months) and longer (5 months) intervals between the second dose and booster in people living with HIV. The longer-interval group had higher neutralizing antibody and seropositivity rates than the shorter-interval group. Interestingly, the longer-interval group showed prolonged immunity in both CD4 count subgroups (< 200 and ≥200 cells μL^−1^) and had a higher seropositivity rate than the shorter-interval group at 6-month follow-up. However, the neutralizing response to the Omicron BA.5.2 subvariant was inadequate in all groups, including the healthy control. The longer interval between doses was shown to be useful for boosting immunity. On the contrary, the interval period may increase the likelihood of severe infection if individuals become infected due to waning immunity, especially in immunocompromised subjects. Barkhordar et al. conducted a clinical trial assessing the homologous prime-boost vaccination of the SARS-CoV-2 RBD-Tetanus toxoid conjugated (RBD-TT-conjugated) vaccine in acute leukemia with allogeneic hematopoietic stem cell transplantation. AEFIs were mostly local reactions, with no serious AEFIs reported. The booster could substantially increase immunity compared to the pre-booster. The other two cohort publications also focused on immunocompromised patients. Gaete-Argel et al. compared mRNA booster responses in solid organ transplant recipients receiving full priming inactivated or mRNA vaccines. Boosters, whether homologous or heterologous, increased seropositivity rates against SARS-CoV-2 in wild-type and Omicron BA.1 recipients. However, some recipients did not respond to the booster, which is a common problem in the immunocompromised population. Bulnes-Ramos et al. reported that booster vaccination significantly increased immune responses in kidney transplant recipients (KTR), and these responses were positively correlated with thymosin-α1 levels.

The cross-sectional studies had different types of participants. Hossain et al. compared various types of vaccination among Bangladeshi migrant workers. The booster vaccination group, whether naïve or convalescent, exhibited significantly higher antibody levels compared to the non-booster group. As in other studies, the mRNA platform had higher antibody levels than other platforms. Al-Rifai et al. evaluated humoral and cellular immunity in various COVID-19 vaccine types and showed that boosters enhanced immunity compared to full priming vaccination alone. During the Omicron predominant wave, Yang et al. used data from a large hospital in Shanghai, China, during the Omicron BA.2 sub-variant wave and revealed that viral RNA was rapidly cleared in inactivated vaccine recipients, especially in booster recipients compared with unvaccinated individuals. The studies focus on immunocompromised host participants. Pérez-Flores et al. suggested that using mTOR inhibitors may enhance the capacity of the immune system to respond to the booster in kidney transplant recipients. Additional clinical trials are recommended to confirm this concept. Feng et al. focused on inflammatory bowel disease (IBD) patients in Shanghai, China, during the Omicron BA.2 and BA.2.2 sub-variants wave. The vaccination rate, including booster doses, in IBD patients was lower than in asymptomatic carriers and healthy individuals, with one-third of the unvaccinated citing fear of IBD exacerbation as the reason for refusing vaccination. However, reactogenicity was not significantly different between IBD and healthy individuals. Xu Y. et al. delineated vaccination status, reactogenicity, and perceptions among Chinese breast cancer survivors [Three studies focus on immunocompromised]. Unvaccinated individuals feared disease progression or interference with treatment, while vaccinated individuals were primarily concerned about infection or workplace requirements. Side effects were acceptable in the vaccinated group, suggesting the need to promote vaccination and raise awareness of vaccine safety among cancer patients to increase vaccination rates.

There are several population-based studies in this Research Topic. Montes-González et al. assessed nationwide surveillance in Mexico, focusing on the hybrid immunity against re-infection and severe disease during the Omicron-predominant circulation wave. This study suggested that hybrid immunity could significantly reduce the risk of re-infection and severe infection compared to unvaccinated convalescents. Moreover, the heterologous booster could reduce the risk of re-infection and severe infection compared to the homologous booster strategy. Zhou C. et al. comprehensively assessed the case fatality rate among booster recipients in 32 countries with multi-dimensional explanatory variables. Boosters were identified as a crucial factor in reducing the age-adjusted case fatality rate. The study also identified different risk factors at the country level. Matveeva and Shabalina analyzed data from 29 European countries and found that slower vaccination rates, including delayed booster administration, were associated with higher excess fatalities from COVID-19. Vaccine protection was highest during the Delta predominant circulation wave and decreased during the Omicron BA.1/BA.2 sub-variant predominant circulation wave. However, additional booster doses were found to be beneficial in preventing excess deaths during the Omicron wave.

Four systematic reviews are also included in this Research Topic, two of which focus on immunocompromised hosts. Sun et al. reviewed vaccine response and safety in cancer patients, indicating that vaccines are generally safe and well-tolerated, with mild reactogenicity. However, the seroconversion rate after the second dose is insufficient for all participants. Booster doses are critical to increasing seropositivity immunity, given the higher risk of severe infections in cancer patients. Martinelli et al. reviewed the fourth dose of COVID-19 vaccination (second booster) in immunocompromised recipients, including oncology patients, organ transplant recipients, CAR-T cell therapy, autoimmune disorders, and HIV infection subjects. This review focused on the humoral response, efficacy, and safety. The booster enhanced the humoral immune response. No serious AEFIs were reported. One study focused on older adults, with Xu K. et al. analyzing data from randomized control trials in this population (aged ≥60 years). Vaccination significantly reduced hospitalization, including ICU admission, and death in older adults. Booster doses notably elevated the geometric mean compared to full priming or partial vaccinations. Local reactions occurred more often than systemic reactions, and serious AEFIs were rare. The final study focused on adults: Xu J. et al. analyzed data from cohorts or randomized control trials that focused on booster vaccination. The booster, either homologous or heterologous, could enhance both humoral and cellular immune responses. Booster doses significantly reduced the risk of infection, including in severe conditions, in addition to ICU admission and death.

In summary, preclinical studies of novel vaccine candidates are promising. Multi-T cell epitope (MTE) stimulation could potentially prevent life-threatening disease, regardless of the antibodies presented. The live attenuated bacterial vector could be a possible future route to stimulate mucosal immunity, the first barrier before the viral invasion of the host, through needleless administration. Heterologous strategies with different antigens from VOCs could stimulate a broader spectrum against the virus. Most articles focused on immunogenicity from cross-sectional or longitudinal studies, which are easier to conduct and measure with straightforward outcomes. Continuous outcomes from immunological assessments require smaller sample sizes than dichotomous outcomes. Such immunological outcomes are likely to provide useful data for decision-making. All participants benefited from booster vaccination, which could substantially increase immunity and cross-reactivity to the VOCs. However, there remains controversy surrounding immunological assessments, particularly concerning “immunobridging,” especially with new variants that reduce vaccine efficacy by evading immunity. The systematic reviews included here prove that booster vaccination enhances immunity and could protect booster vaccination recipients from life-threatening infections and fatalities more than non-booster vaccination recipients. Furthermore, big data analysis from European countries revealed that delaying booster vaccination was linked to higher excess deaths.

In conclusion, the current data support the benefits of booster vaccination over non-vaccination, particularly during a crisis. However, it's important to note that no intervention, including fully FDA-approved drugs or vaccines, is entirely risk-free. Any vaccination or treatment should be discussed with a healthcare professional to weigh the associated risks and benefits.

## Author contributions

RY: Supervision, Writing – original draft, Writing – review & editing, Conceptualization. KA: Writing – original draft, Writing – review & editing. AR: Writing – review & editing.
